# Ethinylestradiol_30μg_-drospirenone and metformin: could this combination improve endothelial dysfunction in polycystic ovary syndrome?

**DOI:** 10.1186/1472-6823-12-9

**Published:** 2012-06-19

**Authors:** Ioana Rada Ilie, Ioan Marian, Teodora Mocan, Razvan Ilie, Lucian Mocan, Ileana Duncea, Carmen Emanuela Pepene

**Affiliations:** 1 Department of Endocrinology, University of Medicine and Pharmacy, 3-5 Louis Pasteur, 400349, Cluj-Napoca, Romania; 2 Department of Internal Medicine-Cardiology, University of Medicine and Pharmacy, Cluj-Napoca, Romania; 3 Departmen of Physiology, University of Medicine and Pharmacy, Cluj-Napoca, Romania; 4 Department of Obstetrics and Gynecology, University of Medicine and Pharmacy, Cluj-Napoca, Romania; 5 3-rd Department of Surgery, University of Medicine and Pharmacy, Cluj-Napoca, Romania

**Keywords:** Ethinylestradiol_30μg_-drospirenone, Flow-mediated dilatation, Endothelial dysfunction, HsCRP, Metformin, Polycystic ovary syndrome

## Abstract

**Background:**

We are hereby investigating for the first time the effect of the association ethinylestradiol_30μg_-drospirenone _3mg_ (DRP/EE_30μg_) plus metformin and weight loss on endothelial status and C-reactive protein (hsCRP) levels in polycystic ovary syndrome (PCOS).

**Methods:**

25 young women with PCOS (mean age 22.76 ± 0.83 years, body mass index (BMI): 28.44 ± 6.23) who completed the study were prospectively evaluated. The oral contraceptive- DRP/EE_30μg_ (21 days/month) and metformin (1700 mg daily) were administered for 6 months to the PCOS group. Additionally, the 15 overweight and obese patients (BMI > 25 kg/m2) were instructed in a diet of no more than 1500 cal daily. Primary outcome measures were surrogate markers of cardiovascular disease and included endothelial function, *i.e.* flow-mediated dilatation (FMD) on the brachial artery and endothelin-1 levels, as well as hsCRP concentrations, body composition (measured by whole-body dual-energy X-ray-absorptiometry) and insulin resistance. Variables were assessed at baseline, as well as after our medical intervention.

**Results:**

The combination between DRP/EE_30μg_ plus metformin combined with weight loss triggered a significant improvement in the FMD values (FMD-PCOS_basal_ 3.48 ± 1.00 *vs* FMD-PCOS_6 months_7.43 ± 1.04, p = 0.033), as well as body composition and insulin insensitivity (p < 0.05). Regarding hsCRP levels, there was no significant intragroup (PCOS_6months_ – PCOS_basal_) difference.

**Conclusion:**

A 6-month course of metformin- DRP/EE_30μg (_associated with weight loss) improves the endothelial dysfunction in PCOS and shows neutral effects on hsCRP concentrations as an inflammation marker. These data demand for reevaluation of the medical therapy in PCOS, particularly in women with additional metabolic and cardiovascular risk factors (ClinicalTrials.gov Identifier: NCT01459445)**.**

## Background

Women with polycystic ovary syndrome (PCOS) frequently cluster several cardiovascular risk markers and early subclinical atherosclerosis which seem to be in relation to their unfavourable endocrine and metabolic milieu [1,2] represented mainly by insulin resistance, central obesity and hyperandrogenemia [3]. Endothelial dysfunction and arterial stiffness are considered early stages of cardiovascular disease development [4]. Previous studies [3,5-7], have shown that endothelial function is impaired in women with PCOS. Moreover, low-grade chronic inflammation plays a major role in inducing endothelial injury, atherosclerosis development and progression in these patients.

Lifestyle modification, diet and weight loss are essential for the cardiovascular disease and diabetes mellitus prevention in overweight and obese PCOS women. In addition to these, and aside from therapeutic strategies directed towards fertility restoration, the current pharmacological treatment of this disease is mainly based on two drug categories: (combined) oral contraceptives ((C) OC) and insulin sensitizers.

The insulin sensitizer metformin has been shown to ameliorate insulin resistance, reduce hyperandrogenism and triglyceride (TG) levels in PCOS [7-9]. Moreover, some studies also reported endothelial structure and function improvement [7,10] and a decrement of highly sensitive C-reactive protein (hsCRP) concentrations in PCOS after metformin [11,12].

On the other hand, COC have a long history of use in these patients and are prescribed for obtaining a regular menstrual cycle and for improving hyperandrogenism. Because COCs might adversely influence insulin resistance, glucose tolerance, lipid profile [9,11] or aggravate chronic inflammation [11,13] the possibility of worsening the already unfavorable cardiovascular risk profile of PCOS subjects is of concern. However, the metabolic effects of pills are extremely variable; for example estrogens impair insulin action dose-dependently and the associated progestins may modify these effects [14]. It was suggested that when a dose of ethinylestradiol (EE) < 50 μg/day is used, the effects of the COCs on the lipid and glycoinsulinemic metabolism is related to the progestin used in the combination [15]. Drospirenone (DRP) is a progestin with antiandrogenic and antimineralocorticoid activity. However, the studies assessing the effect of the COC containing 30 μg EE + 3mg DRP (DRP/EE_30μg_) on surrogate markers of atherosclerosis are few and inconclusive [15,16]. All the hereinabove considered, the present study aimed to assess the effects of DRP/EE_30μg_ combined with metformin and weight loss by means of dietary intervention on indices of endothelial dysfunction, *i.e.* flow-mediated dilation (FMD) and serum endothelin-1 (ET-1), serum hsCRP and lipids, and insulin resistance in young women with PCOS.

## Methods

### Study populations

We prospectively studied 26 women with PCOS who presented to our clinic. The study protocol was conducted with the approval of the local ethics committee (246/09.02.2011), in accordance with the Helsinki Declaration. Participants gave written informed consent before their inclusion in the study protocol. The diagnosis of PCOS was based on Androgen Excess Society 2006 guidelines [17]. Hyperandrogenism was defined as hirsutism and/or as an elevated total testosterone concentration. Menstrual irregularities were defined as oligomenorrhea (eight or fewer menses/ year) or amenorrhea (absence of menstruation for 3 consecutive months). Polycystic ovaries were defined by the ultrasound appearance of 12 or more follicles in each ovary measuring 2–9 mm in diameter and/or ovarian volume greater than 10 ml. Secondary causes of hyperandrogenism such as hyperprolactinemia, thyroid disease, androgen-secreting tumours, Cushing’s syndrome and congenital adrenal hyperplasia were excluded in all patients. The presence and extent of hirsutism were quantified using the Ferriman-Gallwey (F-G) score. Exclusion criteria included current or previous use (within 6 months) of oral contraceptives, anti-androgens, ovulation induction medications, drugs known to affect carbohydrate-lipid metabolism, or a personal history of diabetes.

### Study design

At the first visit, subjects with PCOS and BMI < 25 kg/m2 and those with BMI > 25 kg/m2 were instructed in a diet of no more than 2000- and respectively 1500 cal daily, composed of 50% carbohydrates, 20% proteins, and 30 % fat –with a polyunsaturated-saturate ratio of 2:1. Dietary adherence was assessed by review with the same investigator (I.I.) at 3 and 6 months after inclusion in the study. The treatment with the monophasic COC- DRP/EE_30μg_ (21 days/month) and metformin, given as 850 mg twice per day, was started the fifth day of a spontaneous or progestin-induced menstrual cycle. To reduce metformin gastrointestinal side effects, doses were titrated up over a 20-day period starting at 425 mg up to a final dose of 1700 mg /day (two tablets daily, one before lunch and one before dinner). All women received their medications for 6 months.

A complete history and physical examination, including BMI and waist hip ratio (WHR) were determined by the same physician. Systolic blood pressure (SBP) and diastolic blood pressure (DBP) were measured in the right arm, with the subjects in a seated position, using a mercury sphygmomanometer. More than 5 cigarettes/ day was considered active smoking, while a sustained physical activity (fitness, swimming, jogging etc.) of at least 1h per day, at least 3 times a week, for at least 3 consecutive months was considered significant. None of the study subjects performed significant physical activity before or during the study. All patients were instructed to not modify their physical activity throughout the trial.

PCOS subjects were investigated at baseline, during early follicular phase (days 2–5) of a spontaneous or progestin-induced menstrual cycle, and after six cycles of treatment-during days 5–7 of week 4 (week of placebo pills, no exogenous hormones). Evaluation included anthropometric, laboratory, flow-mediated dilation (FMD) and body composition measurements. In seventeen PCOS subjects (68%) menstruation was induced using dydrogesterone, 20 mg/day for 5 days, a progestin which appears to exert neutral effects on nitric oxide release, as recently demonstrated [18].

### Assay methods

All blood samples were obtained between 08.00 and 10.00 h in the morning after overnight fasting. Blood samples were immediately centrifuged and the serum obtained was stored at −80°C until the time of assay. Serum fasting glucose (GLU, mg/dl) was measured by the glucose oxidase colour method (Glucose GOD/PAP; Diagnosticum Zrt, Budapest, Hungary). Total cholesterol (TC, mg/dl) and TG (mg/dl) were measured by an enzymatic, colorimetric method (Diagnosticum Zrt, Budapest, Hungary).

All other measurements were performed using the ELISA TECAN auto-analyzer. Insulin (INS, μU/ml), total testosterone (TT, ng/ml), sex hormone-binding globulin (SHBG, nmol/l), endothelin-1 (ET-1, pg/ml) were all measured using commercial enzyme–linked immunosorbent assay kits from DRG Instruments, Marburg, Germany. The intra-assay coefficients of variation for low and high values for INS, TT, SHBG and ET-1 respectively were as follows: 2.6% and 1.8%, 4.1% and 3.3%, 8.6% and 5.3%, 8.8% and 6.7% respectively. The intra-assay coefficients of variance for hsCRP were 6. 9% for CRP values <1.0 mg/l and 4.1% for CRP values >3.0 mg/l, respectively. The minimal detectable concentrations for ET-1 and hsCRP were 0.41 pg/ml and 0.02 μg/ml.

The free androgen index (FAI) was calculated according to the equation: FAI (%) = TT (ng/ml) x 3.47 X 100/SHBG (nmol/l). Insulin resistance was estimated by the homeostasis model assessment of insulin resistance (HOMA-IR) defined fasting glucose (mg/dl) x insulin (μU/ml)/405 and by the quantitative insulin sensitivity check index (QUICKI) defined as 1/ [log (fasting insulin) + log (fasting glucose)].

### Body composition

Body composition was assessed by whole-body dual-energy X-ray-absorptiometry (DXA) with a DPX-NT (GE, Madison, USA) device. Fat-free mass was automatically determined as the difference between total body weight and bone mineral content and fat mass. Both total and segmental fat mass and fat-free mass were expressed as weight percentage. The coefficient of variance, evaluated at 3% for total fat mass, was determined by measurements on 10 patients, each one evaluated 3 times.

### Hemodynamic studies

FMD was measured for all subjects by the same cardiologist, who was blinded to PCOS status, using a colour Doppler (AGILENT SONOS 4500) with a high-resolution 10-Mhz linear probe. Each patient was taken into a quiet, temperature-controlled room at 20-25°C. After resting in a supine position for 15 minutes, the right brachial artery was identified and its position marked at about 5 cm above the elbow joint. Diameter (mm) of the artery was measured at end-diastole. After the resting measurement, limb flow occlusion was produced by inflating a standard sphygmomanometer cuff on the upper arm to 50 mmHg above systolic pressure for 5 minutes. This caused ischemia and consequently, dilatation of downstream resistance vessels. Subsequent cuff deflation induced a brief high-flow state through the brachial artery (reactive hyperemia) for endothelial nitric oxide release, to accommodate the dilated resistance vessels. The brachial artery was scanned continuously for 90 seconds after cuff deflation and the measurements were performed during the 30–90 seconds interval. The vessel’s diameter was measured at the same point with resting measurement at least twice and the maximal diameter was again defined (diameter during reactive hyperemia). FMD was calculated as the percentage maximum change in vessel size from baseline. The coefficient of variation for repeated measurements of resting arterial diameter was 2.3%.

### Statistical analysis

Prospective sample size estimation was performed based on a pilot study data (n = 10 for each group) which showed FMD levels for before and after treatment measurements of 3.37 ± 6.6 and 7.97 ± 4.77, respectively. For alpha = 0.05 and beta = 0.20, we have calculated a sample size of n = 24 patients to be evaluated before and after treatment. An additional estimative 8% was added to the calculated sample size in order to cover patient loss, resulting in a sample size of n = 26. Analysis was performed on the 25 subjects who completed the study. Results are reported as mean values ± SEM. The distribution of continuous variables was tested with Kolmogorov-Smirnov test. Differences among continuous variables before and after treatment were assessed by Wilcoxon test. For this type of analysis additional adjustments for dichotomous weight loss were done using weight loss thresholds of 5% and 10% respectively. Bivariate correlations were performed calculating the Spearman rho coefficient. P values of <0.05 were considered statistically significant. The analysis was performed using SPSS version 17.0 (Chicago, Il, USA) and MedCalc 8.3.1.1.

## Results

The study initially included all 26 subjects who were eligible after screening. During the follow-up period, 1 patient withdrew from the study because of some moderate gastrointestinal side effects related to metformin and was, therefore, excluded. This resulted in a final number of n = 25 patients (ages: 15–30, mean age 22.76 ± 0.83 years) completing the study.

### Treatment and diet effects on measured parameters

Table 1 illustrates the anthropometric characteristics, the main metabolic and hormonal pattern as well as the endothelial and inflammatory profile of these 25 PCOS patients over the 6 months.

**Table 1:** **PCOS subjects (n=25) characteristics at baseline and at the sixth cycles of treatment**. Data as means ± SEM

**Variables**	**Baseline**	**6 months**	**p-value**
BMI (kg/m^2^)	28.44 ± 1.24	26.51 ± 1.00	<0.001^‡^
WHR	0.83 ± 0.07	0.82 ± 0.01	0.071
Glucose (mg/dl)	85.36 ± 1.43	83.08 ± 1.29	0.192
HOMA-IR	3.67 ± 0.32	2.82 ± 0.23	<0.001^‡^
QUICKI	0.31 ± 0.003	0.33 ± 0.004	0.001^†^
Insulin (μU/ml)	17.26 ± 1.37	13.74 ± 1.11	0.001^†^
TC (mg/dl)	198.00±4.87	215.28 ± 6.42	0.005^†^
TG (mg/dl)	100.56 ± 6.82	125.40 ± 12.88	0.04*
Total fat mass (%)	44.08 ± 1.54	42.56 ± 1.47	0.018*
Trunk fat mass (%)	45.44 ± 1.81	44.02 ± 1.81	0.065
Lean mass (kg)	39.03 ± 1.38	37.84 ± 1.32	0.009^†^
TT (ng/ml)	0.84 ± 0.06	0.68 ± 0.02	0.007^†^
FAI (%)	9.70 ± 1.72	3.55 ±1.02	<0.001^‡^
SHBG (nmol/l)	44.41 ± 5.34	148.44 ± 16.22	<0.001^‡^
F-G score	9.72 ± 1.06	8.20 ± 0.92	0.02*
SBP (mmHg)	106.20 ± 2.43	104.00 ±2.39	0.184
DBP (mmHg)	70.80 ± 1.95	72.60 ± 1.68	0.318
hsCRP (mg/l)	5.02 ± 0.72	5.20 ± 0.87	0.518
FMD (%)	3.48 ± 1.00	7.43 ± 1.04	0.033*
Baseline artery diameter (mm)	3.14 ± 0.07	3.03 ± 0.06	0.039*
ET-1 (pg/ml)	2.10 ± 0.59	1.60 ± 0.26	0.179

PCOS, polycystic ovary syndrome; BMI, body mass index; WHR, waist-to-hip ratio; HOMA-IR, homeostasis model assessment of insulin resistance; QUICKI, quantitative insulin sensitivity check index; TC, total cholesterol; TG, triglycerides; TT, total testosterone; FAI, free androgen index; SHBG, sex-hormone binding protein; SBP, systolic blood pressure; DBP, diastolic blood pressure; FMD, flow-mediated dilatation; ET-1, endothelin-1.

*P<0.05;^†^*p<0.01;*^‡^*p<0.001.*

The BMI, total fat mass %, lean mass, fasting insulin and HOMA-IR, the F-G score and both TT and FAI were significantly decreased by the pharmacologic approach and diet in the total PCOS group, while WHR and trunk fat mass % declined only borderline. We also noted a significant increment of QUICKI and SHBG concentrations as well as of TC and TG levels over the study.

The combination of diet with metformin + DRP/EE_30μg_ was associated with a higher FMD (Table 1). A trend towards lower ET-1 levels was found after 6-month DRP/EE_30μg_ and metformin therapy. However, the difference failed to reach statistical significance. Moreover, there was no change in hsCRP levels at the end of the follow-up period as compared to baseline ones (Table 1).

HsCRP levels were significantly correlated with BMI (r = 0.554, p = 0.004), TC (r = 0.600, p = 0.002), total fat mass % (r = 0.484, p = 0.014) and trunk fat mass (r = 0.404, p = 0.045) at the beginning of the research whereas at 6 months they significantly correlated with BMI (r = 0.550, p = 0.004), trunk fat mass (r = 0.431, p = 0.032), insulin (r = 0.418, p = 0.038), HOMA-IR (r = 0.411, p = 0.041), QUICKI (r = −0.412, p = 0.041), and TC concentrations (r = 0.403, p = 0.046) but only borderline with total fat mass (r = 0.353, p = 0.091). No other significant correlation between the studied parameters was found.

### Influence of obesity and smoking habits on post-therapeutic results

Overall the PCOS women achieved a 6.61% (−5.04 ± 5.31kg) moderate weight loss, whereas the obese- overweight and non-obese PCOS subgroups undergone a weight loss of 8.78% (−7.53 ± 4.92 kg) and 2.1 % (−1.3 ±3.41 kg), respectively.

Compared with their non-obese counterparts, obese and overweight PCOS women had higher WHR (p = 0.02), DBP (p = 0.017), FAI levels (p = 0.035), fasting glucose (p = 0.009), fasting insulin (p = 0.003), HOMA-IR (p = 0.001), total and trunk fat mass % (p < 0.001), hsCRP concentrations (p = 0.045) and a lower QUICKI (p = 0.001) and SHBG concentrations (p = 0.031). Considering non-obese and obese PCOS subjects separately, the insulin resistance indices, total fat mass and lean mass showed a significant improvement after 6 months of therapy associated with diet, compared to baseline in the sub-group of obese and overweight women. However, none of the endothelial dysfunction or inflammatory markers seemed to have significantly improved (Table 2). In contrast, non-obese PCOS patients only noted significant changes in markers of hyperandrogenemia and a borderline increase in QUICK index. Regarding the lipid profile, TG levels significantly rose in the obese sub-group while TC concentrations were significantly increased at the end of the 6-month period in the non-obese subgroup and borderline in the obese one (Table 2).

**Table 2:** **Hormonal, metabolic, inflammatory and functional endothelial status in obese and non-obese PCOS women pre- and post-medical intervention**. Data as means ± SEM

**Variables**	**Obese PCOS**		**p-value**	**Non-Obese PCOS**		**p-value**
	**Baseline**	**6 months**		**Baseline**	**6 months**	
BMI (kg/m^2^)	32.56 ± 1.13	29.71 ± 0.94	0.001^†^	22.26 ± 0.55	21.72 ± 0.66	0.263
WHR	0.87 ± 0.01	0.86 ± 0.01	0.440	0.77 ± 0.01	0.75 ± 0.01	0.056
Glucose (mg/dl)	88.40 ± 1.62	84.60 ± 3.96	0.117	80.80 ± 1.91	80.80 ± 2.18	0.859
HOMA-IR	4.48 ± 0.42	3.29 ± 0.30	0.002^†^	2.46 ± 0.17	2.10 ± 0.23	0.139
QUICKI	0.31 ± 0.004	0.32 ± 0.005	0.003^†^	0.33 ± 0.003	0.34 ± 0.005	0.074
Insulin (μU/ml)	20.48 ± 1.79	15.85 ± 1.49	0.002^†^	12.43 ± 0.85	10.57 ± 1.11	0.169
TC (mg/dl)	202 ± 20.65	220 ± 37.84	0.057	192 ± 29.24	208.20 ±20.77	0.047*
TG (mg/dl)	103.20 ± 35.41	135 ± 72.67	0.004^†^	96.60 ± 33.48	111 ± 49.69	1.00
Total fat mass (%)	49.51 ± 3.30	47.43 ± 4.36	0.016*	35.94 ± 4.37	35.26 ± 3.71	0.508
Trunk fat mass (%)	51.82 ± 3.56	50.39 ± 4.00	0.173	35.86 ± 5.44	34.46 ± 5.00	0.203
Lean mass (kg)	40.54 ± 8.52	38.72 ± 8.27	0.006^†^	36.75 ± 2.38	36.51 ± 2.56	0.721
TT (ng/ml)	0.86 ± 0.08	0.68 ± 0.03	0.009^†^	0.80 ± 0.09	0.68 ± 0.01	0.241
FAI (%)	12.33 ± 2.64	4.56 ± 1.64	0.011*	5.74 ± 0.82	2.04 ± 0.41	0.007^†^
F-G score	10.40 ± 1.42	8.67 ±1.21	0.007^†^	8.70 ± 1.64	7.50 ± 1.45	0.016*
SHBG (nmol/l)	35.98 ± 6.10	144.03 ± 23.03	0.001^†^	57.05 ± 8.59	155.06 ± 22.53	0.013*
SBP (mmHg)	109.67 ± 3.02	104.67 ±3.29	0.042*	101.00 ± 3.63	103.00 ± 3.59	0.732
DBP (mmHg)	74.67 ± 2.41	74.67 ± 1.91	0.886	65.00 ± 2.35	69.50 ± 2.93	0.098
HsCRP (mg/l)	6.10 ± 0.91	6.88 ± 1.14	0.307	3.40 ± 1.03	2.69 ± 0.95	0.646
FMD (%)	4.57 ± 1.31	8.58 ± 1.29	0.133	1.65 ± 1.44	5.55 ± 1.65	0.161
Baseline artery diameter (mm)	3.15 ± 0.11	3.06 ± 0.09	0.402	3.12 ± 0.08	2.99 ± 0.09	0.779
ET-1 (pg/ml)	2.36 ± 0.92	1.80 ± 0.42	0.198	1.71 ± 0.55	1.32 ± 0.20	0.508

PCOS, polycystic ovary syndrome; BMI, body mass index; WHR, waist-to-hip ratio; HOMA-IR, homeostasis model assessment of insulin resistance; QUICKI, quantitative insulin sensitivity check index; TC, total cholesterol; TG, triglycerides; TT, total testosterone; FAI, free androgen index; SHBG, sex-hormone binding protein; SBP, systolic blood pressure; DBP, diastolic blood pressure; FMD, flow-mediated dilatation; ET-1, endothelin-1;

*P < 0.05; ^†^p < 0.01.

Even after adjusting for a decline of >5% in BMI, there was still a significant difference between the basal values of HOMA-IR (p = 0.014), QUICKI (p = 0.026), INS (p = 0.016), SHBG (p = 0.003), total fat mass % (p = 0.007), trunk fat mass % (p = 0.033), lean mass (p = 0.016) and a borderline one with respect to FMD (p = 0.093) and their values at 6 months. The adjustment for > 10% decrease in body weight was still accompanied by significant differences in pre-treatment vs post treatment values of total fat mass % (p = 0.043) and SHBG (p = 0.028), respectively. Additionally, the unfavorable increments in TG levels over the 6- month treatment observed in our research were significant after applying the same adjustments (p = 0.012, p = 0.027 respectively).

Eight (32%) PCOS women were currently light smokers (i.e. no more than 10 cigarettes per day, <5 years). There were however no statistically significant differences in any of the studied parameters between PCOS smokers and PCOS non-smokers at baseline. Regarding the smoking influence, the fasting glucose (p = 0.021), both HOMA-IR (p = 0.003) and QUICKI (p = 0.006), SHBG levels (p < 0.001) and FAI (p = 0.003) improved significantly in non-smokers during the study and they showed no changes or only borderline modifications towards a better status in smoker PCOS subjects. BMI, insulin levels and F-G score decreased significantly regardless of the smoking status (p < 0.05). Neither FMD nor ET-1 or hsCRP significantly changed during the study as a function of smoking.

## Discussions

We report for the first time that the 6-month treatment with metformin 1700mg/day and the antiandrogenic COC-DRP/EE _30μg_ combined with diet has an overall beneficial effect on endothelial function and shows a neutral effect on hsCRP levels in obese and non-obese women with PCOS.

A novel finding here was the significant improvement of FMD with the medical intervention. However, it is difficult to determine which of the three interventions are responsible for this improvement, especially because when individually assessed, the weight loss, metformin and particularly DRP/EE_30μg_ effects on the endothelial function are not settled. Likewise, several recent studies have demonstrated either the favourable impact of lifestyle modifications such as caloric restriction and increased physical activity in improving endothelial function [19-21] or, on the contrary, no improvement of FMD with weight loss [22,23]. However, in PCOS, metformin seems to have improved endothelial function in both obese and non-obese subjects [7,8,10,11,24,25], although not constantly [9]. FMD appears to increase when circulating levels of estrogens augment naturally or synthetically [26-29] and this beneficial vasodilator effect of estrogens on the arterial function might be antagonized by some certain types of progestins [30,31], but not by DRP [32]. Regarding the DRP/EE_30μg_, there are very few researches investigating its effect on endothelial function and none evaluating the effect in combination with metformin. Therefore, in PCOS the DRP/EE_30μg_ combination administered for 6 months has been shown not to modify the levels of ET-1 which, however, were found to be similar to those of controls and within normal limits [15] or the normal basal vascular reactivity of these women [16]. Others concluded that, while in lean patients with PCOS, the DRP/EE_30μg_ does not seem to affect endothelial function, in overweight PCOS women it does not counteract the loss of weight due to healthier lifestyle changes, which is associated with an improvement of insulin sensitivity and FMD [15]. Of note, FMD did not report a significant improvement when evaluating non-obese and obese PCOS subjects separately and this can be accounted for by the small number of subjects in the two groups.

The present results also show that the combination of metformin + DRP/EE_30μg_ associated with lifestyle changes has overall beneficial effects on body composition and carbohydrate metabolism in the whole study population and in the obese subjects. The milder metabolic abnormalities in non-obese PCOS women (Table 2) and the small number of subjects may explain the lack of significant effect of medical intervention on body composition and the only borderline improvement of insulin sensitivity in these subjects. However, our results are similar to those of Ibanez et al. who demonstrated that a 3-month treatment with metformin 850 mg/day + DRP/EE_30μg_ does not influence the insulin resistance in another group of young non-obese patients with PCOS but with increased adiposity [33]. Nevertheless, the decrease in body weight explains part of our results as a modest weight reduction of 5-10% was demonstrated to be enough to improve insulin resistance [34]. However, we have shown that differences in several parameters persisted after the adjustment for a decrease of > 5% or >10% of body weight thus suggesting that the drug combination used displays an improvement in the insulin sensitivity and can attenuate the central and total body adiposity of young PCOS women. Previous results showed that the increased adiposity found in non-obese adolescents with PCOS diverged further from the norm in subjects on DRP/EE_30μg_ alone [35,36] and that the same OC increased TG levels in both overweight and non-obese women with this disease as well as in young, non-obese but hyperinsulinemic PCOS patients [36-38]. Moreover, in a subsequent research by the same authors, the addition of small doses of metformin- 850mg/day to the treatment with DRP/EE_30μg_ for 3 months failed to attenuate body adiposity of PCOS subjects and was accompanied by a further deterioration of TG and interleukin-6 (IL-6) levels from the norm [33]. In contrast, others observed that DRP/EE_20μg_ + metformin 1500mg/day improved insulin sensitivity, increased HDL-cholesterol concentrations and did not significantly change TG levels in a group of young but non-obese and non-insulin resistant women with PCOS [39]. However, a valid comparison between the study findings of Ibanez et al. and Fruzzetti et al., as well as a further one between their results and ours cannot be performed since the doses of metformin and EE used as well as the basal metabolic profile were different between the populations of these reports. Regarding lipid metabolism, although TG levels rose significantly over the study period in the whole study population as well as in the obese subgroup, most probably as a result of the medical therapy, they remained within the normal range. In other words, it can be hypothesized that metformin clearly does not outperform DRP/EE_30μg_ in increasing TG concentrations. Unfortunately, we did not undertake the analysis of cholesterol fraction in our study. Likewise, we do not know if the increase in the total cholesterol is due to a rise in HDL-cholesterol fraction, or to an increase in LDL-cholesterol or both.

Most published data, yet not all [6,40,41], demonstrate increased levels of hsCRP in women with PCOS [5,12,42,43], which may be associated with increased central fat excess rather than PCOS status per se [41,44]. The change in hsCRP levels in our study was not significant by the end of the 6-month follow-up. However, the dosage of metformin and the duration of our study assured the detection of the effects of the medical intervention on hsCRP levels. Therefore, we can conclude that even though moderate weight loss (6–8 %) definitely conferred significant metabolic benefits, it was not sufficient to improve the low-grade chronic inflammation in the total PCOS group and obese subgroup. The following results at the end of the study are noteworthy: both insulin resistance and body adiposity were still abnormal, particularly in the total PCOS group and the obese sub-group, on the one hand and the hsCRP concentrations correlated with trunk fat mass, BMI, TC and insulin resistance indices on the other hand. Therefore, it appears that a more aggressive therapeutic approach and a greater degree of weight loss may be required to achieve metabolic benefits, such as reductions in insulin resistance and body adiposity and consequent decrease of low-grade chronic inflammation. Sustaining our results, it was shown that a 4–5% weight loss improved lipid, glucose, and insulin profiles in women with and without PCOS, but was not effective in lowering CRP concentrations in PCOS women [45] whereas a 15% weight loss in a 2-yr dietary and exercise intervention study was associated with hsCRP reduction [46]. The results obtained in the non-obese subgroup of PCOS women and also those resulted after the adjustment for a decrease >5 % and 10% in body weight showed that the drug combination used in this study did not affect hsCRP levels. It could be hypothesized that metformin and DRP/EE_30μg_ may have either different or even opposing effects on chronic inflammation that may balance the risk out and neutralize it overall or may both have neutral effects on hsCRP. Our hypothesis is based on previous results showing that metformin either decreased [11-13] or, used in lower doses (1000-1500mg/day) and for shorter period of time (3 months), caused no change in hsCRP [8,47], as well as on the fact that the effect of DRP/EE_30μg_ on chronic inflammation and especially on hsCRP has not been settled. As far as we know, there are no reports evaluating the influence of DRP/EE_30μg_ monotherapy on hsCRP levels in PCOS or in other populations. What has been previously observed, though, is that, DRP/EE_30μg_ further increased the abnormal levels of IL-6 found in young women with hyperinsulinemic hyperandrogenism [36]. Previous reports suggested that metformin may have direct actions on vascular cells [10,48]. Additionally, metformin might decrease angiogenesis via nuclear factor-κB and Erk1/Erk5 pathways by increasing the antiangiogenic trombospondin-1, an adipokine, preferentially produced by visceral adipose tissue and highly expressed in obese insulin-resistant subjects [49]. However, it is uncertain whether metformin exerts direct effects on hsCRP levels or whether its beneficial changes on the concentration of this inflammation marker are only the result of improved glycemia, insulin resistance, abdominal fat excess and weight loss. Complex interactions and mechanisms might be implied. On the contrary, the serum CRP levels have been shown to increase after a 6-month treatment with COC in young overweight and obese women with PCOS, even if the COC contains an anti-androgen [11,13]. Both estrogens and progestin content and dosage appear to be implicated in CRP regulation [50-52], even though the role of oestrogen might be more important than that of progestin [52]. As far as the mechanism of action is concerned, the literature has been sustaining the direct role of COCs in hsCRP determination by affecting the latter’s metabolic and genetic regulation [52]. Hence, a direct estrogen action on the liver was proposed since it is the oral estrogen, and not transdermal estradiol (the latter avoiding the first pass liver effect), which leads to increased serum CRP levels [50,53]. Moreover, COCs have been demonstrated to increment CRP concentrations, without increasing IL-6 ones, suggesting that COCs stimulate hepatocytes to synthesize CRP in a direct way and not via IL-6 mediated inflammation [52]. Regarding anti-androgens, even though their mechanisms of action are not settled, they also appear to modulate and decrease inflammation in PCOS. Hence, low-dose flutamide added to metformin and a fourth-generation OC has been identified to attenuate the hypoadiponectinemia, lean mass deficit as well as central adiposity in young women with PCOS [33]. Up-regulated in states of insulin resistance, IL-6 differentially regulates androgen receptor transactivation via three distinct signaling transduction pathways, the overall effect depending on the balance among these pathways and the androgen concentrations. For instance, at androgen concentration above normal female range and below normal male range, IL-6 and androgens could act synergistically on the androgen receptor [26,33], hereby increasing androgen action. On the other hand, androgen excess in women favors an adipose body composition, including in the abdominal region. A vicious circle amplifying chronic inflammation is thus established. Moreover, adiponectin, which exerts insulin-sensitizing effects, is reversibly down-regulated by androgens and IL-6, but not by estrogens [27]. Therefore, the mechanism of action of flutamide might be at least partly explained by restoration of the androgen receptor transactivation balance and counteraction of the androgen – and IL-6- induced down-regulation of adiponectinemia [33]. Additionally, the mineralocorticoid and the androgen recepto antagonist spironolactone has been shown to inhibit the production of proinflammatory cytokines in patients with congestive heart failure, acting at the transcriptional level and independent of its antimineralocorticoid and antiandrogen activities [28]. We found no data on the effect of the combination metformin-DRP/EE_30μg_ on hsCRP, only that Ibanez et al. showed that the treatment with flutamide-metformin plus DRP/ EE_30μg_ is associated with a consistent fall in CRP and TNF-α levels, especially in patients with the most abnormal values [29].

**In conclusion**, our results suggest that the association metformin- DRP/EE_30μg_ with lifestyle changes has positive impact on clinical and biochemical hyperandrogenemia, carbohydrate metabolism, body composition and endothelial function-increasing FMD and neutral effects on hsCRP levels. ET-1 levels were not significantly altered by DRP/EE_30μg_ and metformin in this follow-up study; however, analyses for ET-1 were significantly underpowered in our set of data. Future revaluation of ET-1 on larger sample size groups is needed for a proper characterization of this marker in relationship with the association metformin- DRP/EE_30μg_ and weight loss in PCOS. Although our study is limited by the lack of a weight loss control group, the results obtained, after adjusting for body weight loss, further suggest that the medical treatment used might have neutral/mild positive effects on hsCRP levels, body adiposity and endothelial function, but it might worsen TG levels. Hence, a thorough assessment of the benefits of metformin- DRP/EE_30μg_ therapy on vascular function require further trials that should include a weight loss group as a control arm. Concerning the subgroup of non-obese PCOS women, the findings of our study suggest that the use of the association metformin- DRP/EE_30μg_ in these women does not seem to affect endothelial function. It can also be considered safe, as no further deterioration of cardiovascular risk factors was noted in this population. Furthermore, the specific effects of the individual components of therapy: metformin and EE/DRP as well as those of the individual COC components on cardiovascular risk markers cannot be ascertained from this study. Nevertheless, these are only cardiovascular risk surrogate markers and we do not know the clinical relevance of our results, which do not provide comprehensive explanations but rather encourage further investigations.

## Abbreviations

(BMI),, Body mass index; ((C) OC), (Combined) oral contraceptives; (DBP), Diastolic blood pressure; (DRP), Drospirenone; (ET-1), Endothelin-1; (EE), Ethinylestradiol; (F-G), scoreFerriman-Gallwey; (FMD), Flow-mediated dilation; (FAI), Free androgen index; (HOMA-IR), Homeostasis model assessment of insulin resistance; (IL-6), Interleukin-6; (PCOS), Polycystic ovary syndrome; (QUICKI), Quantitative insulin sensitivity check index; (SHBG), Sex hormone-binding globulin; (SBP), Systolic blood pressure; (TC), Total cholesterol; (TG), Triglycerides; (TT), Total testosterone; (WHR), Waist hip ratio.

## Competing interests

There is no conflict of interest that would prejudice the impartiality of this scientific work. The authors alone are responsible for the content and writing of the article.

## Authors' contributions

IRI has contributed to the design of this study; she was directly implicated in conducting the research, in collecting, analyzing and interpreting data as well as in editing the manuscript and critically revising it. IM has contributed to the design of this study, the measurement of FMD and the data analysis and interpretation. TM participated in the design of the study and performed the statistical analysis. RI has contributed to the design of this study; data collection and interpretation, gynecological ultrasound examination. LM analyzed the data and acquired funding. ID participated in the design of the study and helped to draft the manuscript. CEP has contributed to the design of this study; she was directly implicated in conducting the research, in interpreting data and editing the manuscript as well as in acquiring funding. All authors read and approved the final manuscript.

## Pre-publication history

The pre-publication history for this paper can be accessed here:

http://www.biomedcentral.com/1472-6823/12/9/prepub
